# Cellulose Nanocrystal Membranes as Excipients for Drug Delivery Systems

**DOI:** 10.3390/ma9121002

**Published:** 2016-12-12

**Authors:** Ananda M. Barbosa, Eduardo Robles, Juliana S. Ribeiro, Rafael G. Lund, Neftali L. V. Carreño, Jalel Labidi

**Affiliations:** 1 Postgraduated Program in Science and Material Engineering, Technology Development Center, Federal University of Pelotas, Gomes Carneiro 1, Pelotas-RS 96010-610, Brazil; ananda.barbosa@ufpel.edu.br (A.M.B.); neftali@ufpel.edu.br (N.L.V.C.); 2 Chemical & Environmental Engineering Department, University of the Basque Country UPV/EHU, Plaza Europa 1, Donostia-San Sebastian 20018, Spain; jerobles001@ehu.eus; 3 Postgraduate Program in Dentistry, Federal University of Pelotas, Gonçalves Chaves 457, Pelotas-RS 96015-560, Brazil; julianar.fo@ufpel.edu.br (J.S.R.); rglund@ufpel.edu.br (R.G.L.)

**Keywords:** cellulose nanocrystals, chlorhexidine, *Staphylococcus aureus*

## Abstract

In this work, cellulose nanocrystals (CNCs) were obtained from flax fibers by an acid hydrolysis assisted by sonochemistry in order to reduce reaction times. The cavitation inducted during hydrolysis resulted in CNC with uniform shapes, and thus further pretreatments into the cellulose are not required. The obtained CNC exhibited a homogeneous morphology and high crystallinity, as well as typical values for surface charge. Additionally, CNC membranes were developed from CNC solution to evaluation as a drug delivery system by the incorporation of a model drug. The drug delivery studies were carried out using chlorhexidine (CHX) as a drug and the antimicrobial efficiency of the CNC membrane loaded with CHX was examined against Gram-positive bacteria *Staphylococcus aureus* (*S. Aureus*). The release of CHX from the CNC membranes is determined by UV-Vis. The obtaining methodology of the membranes proved to be simple, and these early studies showed a potential use in antibiotic drug delivery systems due to the release kinetics and the satisfactory antimicrobial activity.

## 1. Introduction

Flax is a temperate-climate erected plant of the *Linaceae* family with usually 1 m height [1]; flax fibers have been used for thousands of years to make different textile products because of their good performance [2]. Nowadays, flax has been studied like raw material for high value-added nanostructured cellulose production [3]. 

Cellulose nanocrystals (CNCs) correspond to the ordered crystalline components that can be extracted from various cellulosic materials. The extraction of CNCs from fibers usually consists of an acid-induced de-structuring process during which heterogeneous acid hydrolysis involves the diffusion of acid molecules into cellulose fibers, followed by cleavage of glycosidic bonds. This acid hydrolysis step is followed by several cycles of centrifugation, after which dialysis is performed to reach neutral pH and ultrasonication to redisperse CNC in a colloidal water suspension [4,5,6]. Approaches to optimize this methodology have been described [4,5,7].

Properties such as biodegradability, bio-based, and biocompatibility make cellulose an important and fascinating polymer for many applications [8]. Therefore, functional materials can be developed. Recent studies address the cellulose as excipient for drug delivery systems [9,10,11]. The application of CNCs proves to be an alternative that deserves further clarification [12].

This work is aimed to analyze CNCs obtained by high frequency cavitation-inducted acid hydrolysis in order to obtain a different particle with high quality in a shorter period of time than normally used in the conventional hydrolysis and without catalyst or pretreatments and apply the obtained material as a membrane for drug delivery. Different characterizations were performed to assess the CNC and to analyze the colloid solution and the solid-state. 

Furthermore, CNC membranes were developed with the addition of chlorhexidine (CNC-CHX membrane). CHX has broad antiseptic properties against a wide variety of Gram-negative and Gram-positive organisms because the positively charged parts of the CHX molecule react with the phosphate groups of lipopolysaccharides in the bacterial cell wall causing the antimicrobial activity of CHX [13].

CNC-CHX membranes had the antibacterial activities investigated using Gram-positive (G+) bacteria *S. aureus*. *S. aureus* was chosen because this human pathogen can cause problems from skin infections to life-threatening health conditions [14]. The effects of different CHX dosages on the antibacterial activity of the nanocomposites were investigated. 

## 2. Experimental Procedure

### 2.1. Materials

Flax (*Linus usitatisimum*) was kindly provided by Artic Fiber Company Ltd. (Kiiminki, Finland), and sulfuric acid was provided by Panreac^®^ (Barcelona, Spain). All other components were used at laboratory conditions and provided by Sigma-Aldrich Spain (Madrid, Spain).

### 2.2. Cavitation-Induced Acid Hydrolysis

Flax fiber delignification was performed as described by Robles et al. [15]. Acid hydrolysis was performed for the bleached cellulose without pretreatments using a 10.2 M solution of H_2_SO_4_ (1:20 w/v) at two different temperatures (50 and 60 °C) inside an Elmasonic Elma S 70 H sonication bath, where cavitations were induced at 37 kHz, and time was varied from 30, 45 and 60 min. All reactions were stopped with cold distilled water (1:3 v/v), non-hydrolyzed fraction was separated with a glass funnel and the filtered fraction was centrifuged three times at 8000 rpm for 10 min and then dialyzed to distilled water until neutral pH was stabilized for further analysis.

### 2.3. Membrane Production

Three different concentrations of CHX were used to produce the membranes. The fraction of CHX (0.015 g, 0.0015 g or 0.00015 g) was dissolved into an appropriate solvent, and then 10 mL of nanocrystal solution was added for each fraction of CHX (selected nanocrystal suspension corresponds to 50 °C/45 min conditions described in Section 2.2), and this mixture continued being stirred and heated at 50 °C for 3 h. Finally, the suspension was poured into aluminum dishes and put into an oven at 50 °C for 20 h.

### 2.4. Characterization

#### 2.4.1. Raw Material Chemical Composition

Chemical analysis of the flax fibers was performed to determine the amount of cellulose available for further extraction. This characterization was carried out according to the Technical Association of the Pulp and Paper Industry (TAPPI) standard methods. 

#### 2.4.2. Infrared Spectroscopy (FTIR-ATR)

Infrared spectra were recorded on a Spectrum Two FT-IR Spectrometer manufactured by Perkin Elmer, Inc. (London, UK) equipped with a Universal Attenuated Total Reflectance accessory with an internal reflection diamond crystal lens. The defined range was from 800 to 4000 cm^−1^ and the resolution 8 cm^−1^. For each sample, 10 scans were recorded.

#### 2.4.3. Gravimetric Analysis

Cellulose nanocrystals were dry measured to control the yield of each treatment after hydrolysis by using the following equation:

Y = M_C_·M_T_/M_S_·M_O_, (1)

where M_C_ is the mass of dried cellulose nanocrystals (10–15 min in an oven at 105 °C); M_T_ is the mass of the total suspension, M_S_ is the mass of the suspension sample before drying; and M_O_ is the cellulose mass before hydrolysis. 

#### 2.4.4. Size Measurement and Zeta Potential

Particle size and zeta potential were measured inside Malvern Z Nanosizer Equipment (Worcestershire, UK), refractive index for cellulose was considered at 1.47, and measurements were performed at 25 °C. For size analysis, 0.1 g·L^−1^ of cellulose suspension was put inside a disposable plastic cuvette performing 13 scans with an incidence angle of 173° repeated three times for each sample. For zeta potential, nanocrystal suspensions were put inside Malvern folded capillary zeta cells and measured using the Smoluchowsky model (κ • a = 1.50). Ten scans were performed, and three specimens of each sample were measured [16]. 

#### 2.4.5. Nuclear Magnetic Resonance (NMR)

The ^13^C CP/MAS NMR spectrometry was performed using a Bruker 500 MHz spectrometer (Karlsruhe, Germany) at a frequency of 250 MHz with an acquisition time of 0.011 s at room temperature. The spectrum was recorded over 32 scans, and water was used as a solvent for all of the nanocrystalline celluloses.

#### 2.4.6. X-ray Diffraction (XRD)

X-ray powder diffraction was measured to compare crystallinity achieved after each hydrolysis. Patterns were collected with a Panalytical Philips X’Pert PRO multipurpose diffractometer (Almelo, The Netherlands), with samples mounted on a zero background silicon wafer fixed in a generic sample holder, using monochromatic CuKα radiation (λ = 1.5418 Å), 2θ Bragg angle between 5 and 50°, step size of 0.026° and time per step of 80 s at room temperature. Crystalline contributions to the main signal were determined with the intensity observed at the traditional crystalline peaks: −110, 110, 200 and 004, while the amorphous contribution that is attributed to the broad signal observed at 2θ ≈ 18° [17]. Different approaches to estimate the crystallinity used first consisted of the traditional Segal method [18] expressed in Equation (2):

CrI = 100(I_200/I_200 + I_am).(2)

The Segal method is not the most accurate method to determine cellulose crystallinity, but as it is the most widely used, it can be useful to compare results from this study to those made in the past [19,20,21]. As a second and more precise method, Voigt functions were used to deconvolute peaks and determine the integrated surface corresponding to the peaks of 1–10, 110 and 200 Miller indices. Apparent crystallinity was estimated with the following equation:

CrI = 100(A_1–10_ + A_110_ + A_200_)/(A_tot_), (3)

in which A_1–10_ + A_110_ + A_200_ corresponds to the area of the crystalline region and A_tot_ corresponds to the total area [22,23]. Crystallite size was estimated with the Scherrer equation using the peak corresponding to the 200 plane as seen in Equation (4):

d_200_ = κλ/H_200_·cosθ, (4)

with κ being the Scherrer constant most adjusted to the nanocrystal shape (0.86), λ the wavelength (1.5418 Å), H_200_ corresponds to the full width at half maximum intensity (FWHM) and θ is half the Bragg angle at peak maximum given in radians [24,25]. 

#### 2.4.7. Atomic Force Microscopy (AFM)

Atomic force microscopy images were obtained operating in tapping mode with a NanoScope IIIa, Multimode TM-AFM from Digital Instruments-Veeco scanning probe microscope (Plainview, NY, USA) equipped with an integrated silicon tip cantilever with a resonance frequency of 300 kHz. To obtain representative results, different regions of the samples were scanned. 

#### 2.4.8. Release Study

The absorbance of the sampled medium was measured using a UV-Vis V-630 spectrophotometer (Jasco Inc. (Tokyo, Japan)). Firstly, an initial scanning was performed to determine the wavelength at which the readings would be carried out and the length of wavelength of 360 nm as selected. Then, various dilutions were performed obtaining several different concentrations. These solutions of different concentrations were then taken to the UV spectrophotometer at wavelength 360 nm, the absorbance of the solutions at various concentrations was then determined, and then the calibration curve was developed. The drug loaded membranes were weighed (0.0030 g for each sample) and put in a glass vessel containing 10 mL of Phosphate-buffered saline (PBS) buffer solution (pH 7) and the analysis was performed every half hour for four hours, and then readings were taken at 24 and 48 h. The experiments were conducted in duplicate.

#### 2.4.9. Antibacterial Activity

##### Characterization of the Standard Drug

To characterize the chlorhexidine, the minimum inhibitory concentration (MIC) and minimum bactericidal concentration (MBC) were carried out by using broth microdilution techniques as described by the Clinical and Laboratory Standards Institute (M11-A8) with few modifications.

##### Inoculum

*S. aureus* ATCC 19095 cultivars were individually grown overnight in aerobic conditions at 37 °C on Brain and Heart Infusion Agar (BHI), supplemented with 10% sucrose, for 24 h. Colonies of microorganisms were then suspended in Brain and Heart Infusion broth for *S. aureus*, in order to make a suspension of 3 × 108 CFU mL^−1^. The microbial cell turbidity was then adjusted by spectrophotometry (spectrophotometer, Quimis, Brazil) at 405 nm. 

##### Determination of MIC 

The stock solution of chlorhexidine was prepared in a dimethyl sulfoxide (DMSO) solution at a concentration of 500 μg·mL^−1^. Chlorhexidine powder was previously weighed and dissolved in DMSO at 500 μg·mL^−1^ concentration. The solutions were diluted in Muller Hinton medium, and the final drug concentrations ranged from 0.97 to 500 μg·mL^−1^. In addition, 100 μL of the inoculum suspension along with 100 μL of the final product were added to the microculture plate wells. In the negative control, 100 μL of microorganism solution plus 100 μL of Muller Hinton medium was added, and 100 μL of Muller Hinton medium and 100 μL of the final product were added in the positive control. Three replicates were made for each concentration. 

After 24 h of incubation at 35 °C, the absorbance of each well was read on a microplate reader (Thermo Plate TP-Reader, Thermo Fisher Scientific, Waltham, MA, USA) at a wavelength of 492 nm. After the well had been agitated, MIC endpoints were determined as the first concentration of the antibacterial agent at which turbidity in the well was ≥50% less than that in the control well [26].

The measurement for their antibacterial activity, expressed as the percentage of activity (% AE), was calculated with an adaptation from Felício et al. [27] according to Equation (5):

%AE = 100 − (AE − AEB/AC − ACB) × 100,(5)

where AE represents the absorbance of the test plates after the incubation time; AEB is the absorbance of plates containing medium, sample and inoculum at t = 0 of incubation; AC is the absorbance of plates containing negative control (without vehicle) (100% of inoculum growth); and ACB is the absorbance of plates containing culture medium. All of the MIC values were calculated by nonlinear regression.

##### Determination of MBC 

Each inoculum from the previous test that did not show growth was subcultured on agar plates. After 24 h of incubation, the reading was determined by the visible growth of strains. The CBM was considered to be the first concentration of the antibacterial agent at which turbidity in the well was ≥50% less than that in the control well.

##### Antibacterial Activity of the Drug Delivery System

Modified direct contact test was used to evaluate the antimicrobial effect. The modified direct contact test (mDCT) consists of the measurement of cinematic microbial growth by close contact between the micro-organism tested and the material [28] by using microplate 96-well cell cultures. Previously, the samples were sterilized by Gamma radiation into an Eldorado-78 equipment (Atomic Energy of Canada Ltd. (Chalk River, ON, Canada)). After sterilization, the specimens were placed in 96-well plates, with *n* = 6 for each group tested. 

##### Bacterial Strains and Culture Conditions 

*Staphylococcus aureus* ATCC 19095 were individually grown overnight in aerobic conditions at 37 °C on Brain and Heart Infusion Agar (BHI) supplemented with 10% sucrose for 24 h. Colonies of microorganisms were then suspended in Brain and Heart Infusion broth for *S. aureus*, in order to make a suspension of 3 × 10^8^ CFU mL^−1^. The microbial cell turbidity was adjusted by spectrophotometry (spectrophotometer, Quimis, Brazil) at 405 nm. From this inoculum was added 20 μL of bacterial suspension into each well to be evaluated. 

The materials were placed in microplate wells with the aid of sterile forceps being a specimen in each well, and the material was inoculated with 20 μL of microbial suspension (*S. aureus* + BHI + sucralose). The discs were incubated for 1 or 24 h at 37 °C and approximately 100% relative humidity. Microplate wells containing the same volume of bacterial suspension without test discs were also incubated as controls. 

Afterwards, 180 μL of culture medium (BHI, Guangzhou Mecan Trading Co., Ltd., Guangzhou, China) was added into each well and shaken for 10 min. In addition, 100 μL of bacterial suspension from each well was transferred for dilution. Serial dilutions and platings were carried out in disposable Petri dishes containing BHI agar divided into eight parts. Each plate received two drops of 20 μL per dilution and was incubated at 37 °C for 24 h. Beyond the period of incubation, the concentration of colony forming units (CFU mL^−1^) was counted.

## 3. Results and Discussion

### 3.1. Raw Material Chemical Composition

Table 1 presents the chemical composition of flax fibers analyzed with standard methods as the mean of six tests for each standard, as well as a comparison to other previously published works in which flax fibers were used. A high content of cellulose was observed making flax a reliable source for cellulose nanocrystal extraction. The high amount of hemicelluloses present in this fibers makes it desirable to perform an alkali stage in the cellulose extraction sequence, while low lignin content allows for a less aggressive bleaching stage (hydrogen peroxide) to finally extract cellulose with high purity.

### 3.2. FTIR-ATR Spectroscopic Analysis

**Figure 1:**
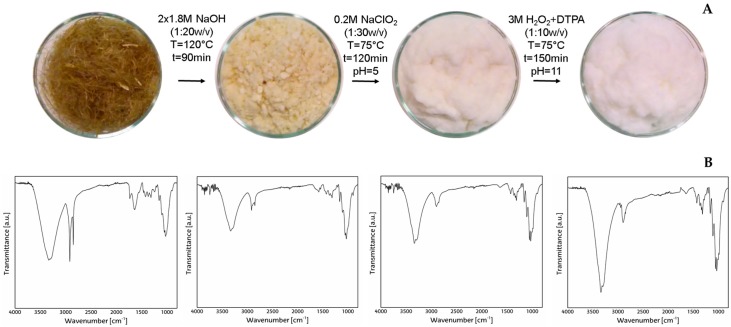
Bleaching sequence diagrams (**A**) along with the corresponding infrared spectrum (**B**).

Flax fibers were received untreated and were pulped and bleached in an integrated Elemental Chlorine Free sequence. In Figure 1A, it is possible to follow the appearance of the flax during the different steps of the treatment. Figure 1B shows the FT-IR spectra of flax fibers as received and flax cellulose fibers. Compared to flax fibers, flax cellulose fibers present more defined peaks and bands that are characteristic to cellulose and can be seen as follows: the band between 3600 and 3000 cm^−1^ corresponds to stretching vibrations of hydroxyl groups in cellulose, and the band between 3000 and 2600 cm^−1^ and 2860 cm^−1^ corresponds to –CH groups, respectively. The signal of 2920 cm^−1^ corresponds to the CH_3_ group and the signal of 2860 cm^−1^ corresponds to the CH_2_ group. The peak at 1645 cm^−1^ can be attributed to the bending mode of the absorbed water in carbohydrates. The band at 1420 cm^−1^ corresponds to CH_2_ bending and the one at 1215 cm^−1^ is originated from the –OH in plane-bending cellulose [31]. The adsorption band at 1150 cm^−1^ can be attributed to C–O antisymmetric bridge stretching. Finally, the peak at 890 cm^−1^ is characteristic of β-glycosidic linkages between glucose units [32].

### 3.3. Colloid Solution Analysis

**Figure 2:**
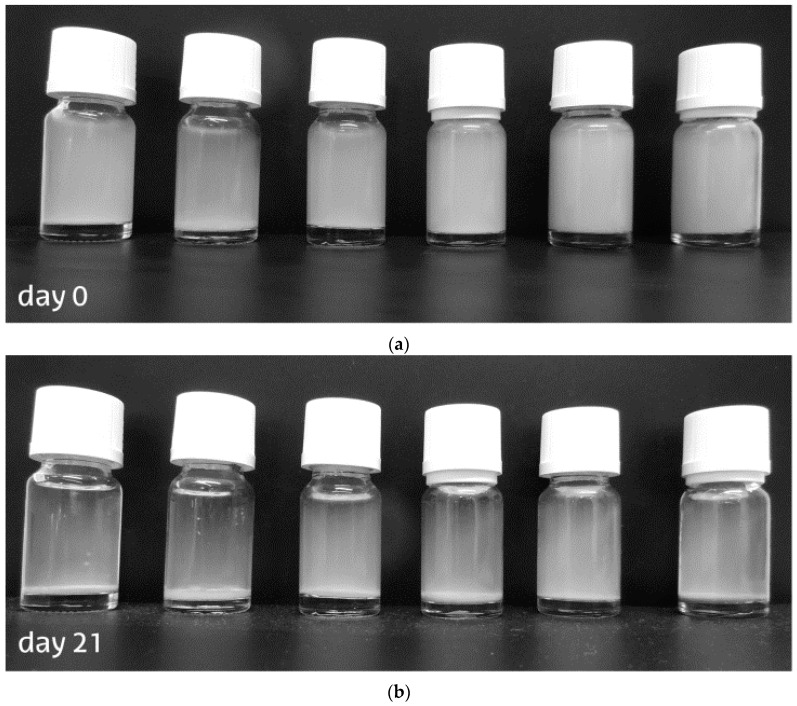
Pictures of the differently obtained cellulose nanocrystals in 1 wt % aqueous suspension before (**a**) and after 21 days of settlement (**b**). (From left to right, 50-30; 50-45; 50-60; 60-30; 60-45; 60-60).

To evaluate colloidal stability of the cellulose nanocrystals, 1 wt % solutions were prepared for each sample, and they were vortexed for a minute and photographed to evaluate initial conditions; solutions were left to rest for 21 days, after which another picture was made (Figure 2). Samples showed different stabilities as colloidal suspensions after the final time elapsed. Anisotropic sedimentation was observed in samples elaborated with lower temperature, while those with higher temperatures had stronger isotropic phases. Regarding sonication time, it is clear that after longer sonication time, particles maintain isotropic dispersion in the water suspension, as the effect of the sulfuric acid after longer time generates a more charged surface of the nanocrystals that acts as a repulsive force maintaining nanocrystals suspended in polar liquids [33]. 

### 3.4. Nuclear Magnetic Resonance (NMR) Analysis

**Figure 3:**
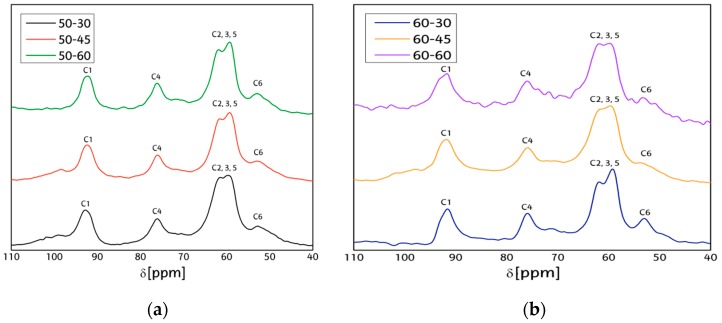
NMR spectra of cellulose nanocrystals after different hydrolysis conditions. (**a**) 50-30 (**black**), 50-45 (**red**) and 50-60 (**green**); (**b**) 60-30 (**blue**), 60-45 (**orange**) and 60-60 (**violet**).

Figure 3 shows the NMR spectra of the CNCs after different hydrolysis conditions, and the main signals between 110 and 55 ppm correspond to crystalline cellulose I [34] and are attributed to carbons in a hydroglucose units listed from C_1_ to C_6_. Differences observed between the CNC are attributed to the differences in the supramolecular structure or the polymer chain packing of the samples, which may be affected during the hydrolysis, and slight variations can be observed in C_1_ for 60-30 and 60-60 samples, with their peaks presenting sharper points in the region closer to C_4_, which is attributed to cellulose Iβ; on the other hand, 50-30, 50-45 and 60-45 celluloses present more rounded peaks for C_1_, which implies a mayor presence of paracrystalline cellulose. In C_4_ for the 60-60 sample, it can be appreciated that the excess of hydrolysis may have disintegrated polymeric chains to main glucose units, thus showing an increment in the amorphous region of C_4_ carbon. 

### 3.5. Size Measurement 

**Figure 4:**
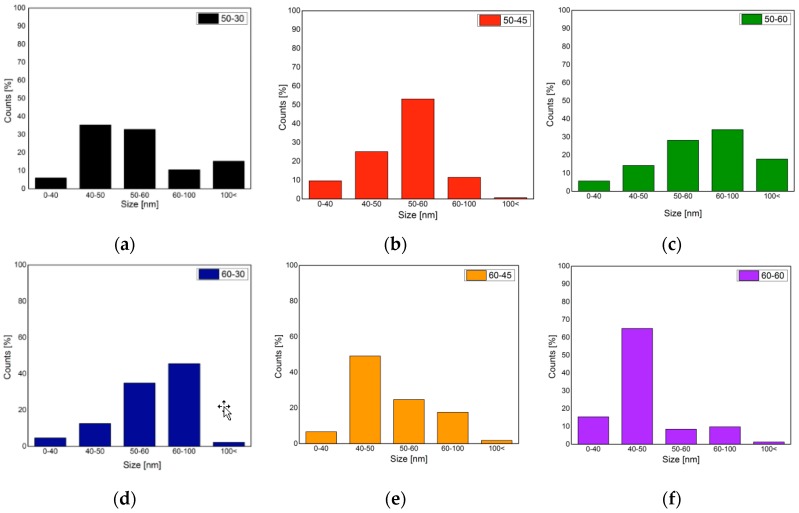
Size histograms of the elaborated CNCs. (**a**) 50-30; (**b**) 50-45; (**c**) 50-60; (**d**) 60-30; (**e**) 60-45; (**f**) 60-60.

In Figure 4, the histograms of the size distribution of each obtained nanocrystal are presented, 60-60 CNCs present the highest homogeneity, as almost 70% of them are contained in the 40–50 nm range, while 50-30 and 50-60 present more even dispersions. The higher percent of CNCs under the 60 nm limit that can be seen in 50-30 can be due to low hydrolysis during the reaction as they have the slowest yield (Table 2), meaning that most of the fibers were not hydrolyzed during the reaction. In addition, 50-45 and 60-45 also present higher homogeneity in their size distribution, as more than half of their counts are in the same range, being 50–60 nm for 50-45 and 40–50 nm for 60-45. These two samples also presented higher yields in general, and CNC yields maintain a close correlation depending on the temperature and time used for the reaction, with the 60-60 treatment being an exception, as it presents smaller particles but also lower yield. This can be due to cellulose polysaccharide chain degradation to single glucose chains.

### 3.6. Yield and Zeta Potential

As a way to analyze the effectiveness of each treatment performed to obtain cellulose nanocrystals, all of the samples were filtered with a glass Buchner funnel with a sintered glass filter No. 1 to retain un-hydrolyzed fibers before centrifugation; therefore, homogeneous cellulose nanocrystal suspensions were obtained, with the removal of larger particles through filtration and the undesirable particles with the centrifugation, and yields were measured gravimetrically and results are shown in Table 2 as well as the zeta potential and the Scherrer approximated. Regarding zeta potential, all of the hydrolyzed nanocrystals showed surface charges in the range characteristic of such particles with only 50-30 and 60-60 being within the moderate range (±30 to ±40 mV), while the rest is in the incipient stability range (±10 to ±30 mV) with no significant variations between them. 

### 3.7. X-ray Diffraction (XRD)

**Figure 5:**
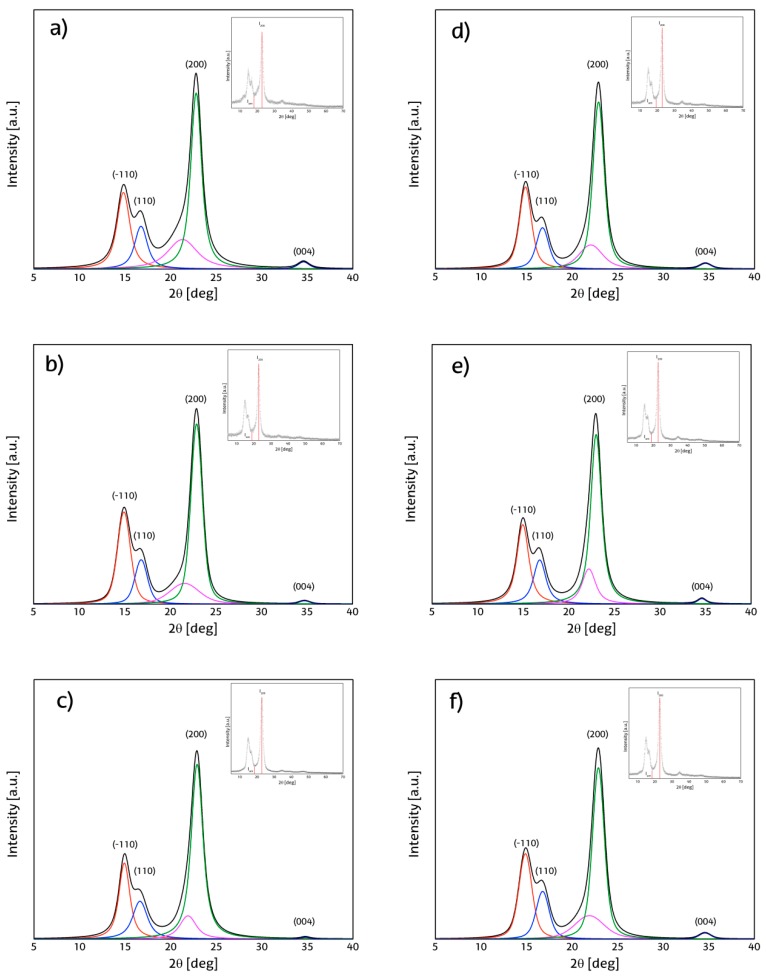
Raw and simulated powder diffraction patterns for: (**a**) 50-30; (**b**) 50-45; (**c**) 50-60; (**d**) 60-30; (**e**) 60-45 and (**f**) 60-60.

Figure 5 shows the scattered raw powder diffraction patterns, the position of the peak associated by the Segal method to main crystalline contribution, and the correspondences to the crystalline plane with Miller index (200) as marked, as well as the point associated with the amorphous contribution to the signal. While the intensity of the (200) plane is more or less the same in all cases, the one attributed to the amorphous region presents a constant decrease at longer reaction times. 

To determine the position of signals generated by the cellulose nanocrystals and gather more information about their composition, powder diffraction patterns were simulated with PeakFit 4.12 software by Systat Software, Inc. (San Jose, CA, USA) to fit the original pattern (R^2^ ≥ 0.998) and then deconvoluted using Voigt curves. All patterns correspond to those of native cellulose (cellulose Iα and Iβ) with stronger contributions attributed to cellulose Iβ. 

Cellulose crystallinity has been extensively evaluated by the Segal method, although this method is now considered imprecise and obsolete for quantitative analysis, and it is still used by many researchers around the world and therefore still represents an option for fast qualitative analysis, as it can be easily obtained and there is a larger amount of bibliographic references to compare with [20,35]. On the other hand, the integrated area method has been gathering more attention as it can represent a more realistic approach to analyze the crystalline domains that are present in the cellulose nanocrystals after the extraction of non-crystalline regions [22]. Table 2 presents the crystallinity index corresponding to Segal and integral area methods as well as the size approximation of the (200) crystalline region as proposed by Scherrer for qualitative purposes. In general, a high crystallinity can be observed for all obtained nanocrystals, with a constant increase in cellulose crystallinity, as reaction time is longer for CNC obtained at 50 °C. In both Segal and deconvolution methods, the change in crystallinity for samples treated at 60 °C is very low, and when time elapses up to 60 min, it decreases, showing a possible de-crystallization of the cellulose nanocrystals. Crystallite size approximated by Scherrer equation (δ_200_) does not vary significantly between the different treatments used and are within the range of other reported data [15,36].

### 3.8. Atomic Force Microscopy (AFM)

**Figure 6:**
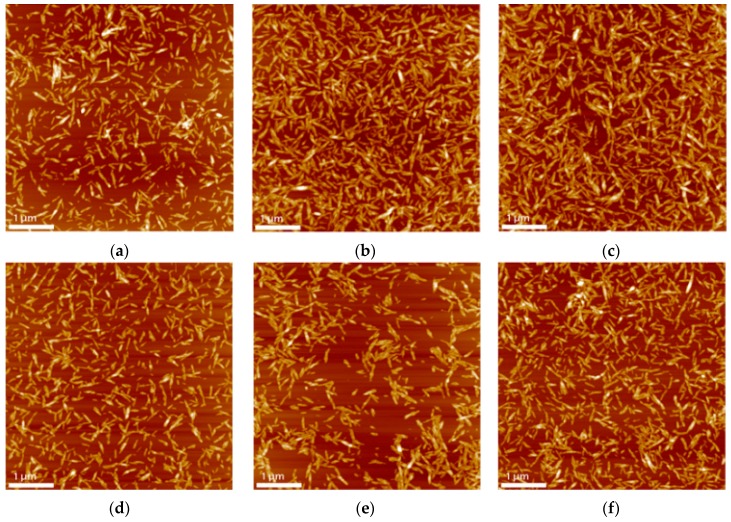
AFM images of cellulose nanocrystals after different hydrolysis conditions. (**a**) 50-30; (**b**) 50-45; (**c**) 50-60 (**d**) 60-30; (**e**) 60-45; (**f**) 60-60.

The obtained nanocrystals present in all cases rod-like morphology characteristics of cellulose nanocrystals and homogeneous size in general (Figure 6), with significant variances depending on the method selected for their obtention. Nanocrystals obtained by methods involving higher temperatures (60-30, 60-45 and 60-60) present smaller particles in both length and width, with 60-60 having the smallest dimensions and 50-60 the largest. In the case of 50-30 nanocrystals, a larger aspect ratio can be observed in atomic force microscopy. 

**Figure 7:**
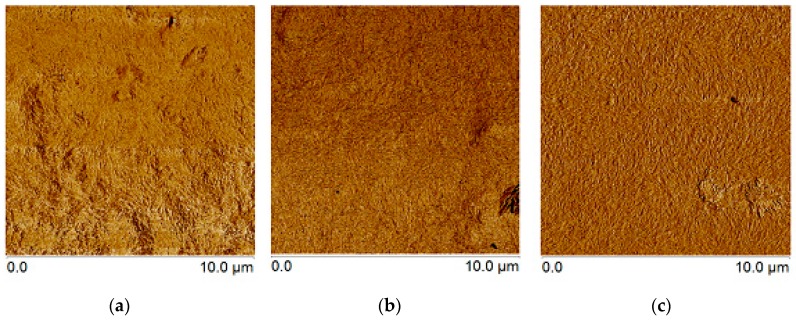
AFM images of CNC membranes + 0.015 g CHX (**a**); CNC membranes + 0.0015 g CHX (**b**); and CNC membranes + 0.00015 g CHX (**c**) and the roughness of the membranes.

The surface of selected elaborated membranes can be observed in Figure 7. The CNC-CHX coatings become evident exhibiting the homogeneity of the membranes with different concentrations of the drug and random orientation. The membrane preparation methodology used in this study favors a controlled release of the drug since the membrane is formed with the drug in contrast to other studies that put the drug at the ready vehicle. The AFM was also used for surface roughness determination; the changes in roughness have the expected behavior, as the amount of drug added reduces the roughness. The lower surface roughness (Table 3) of the samples CNC + 0.0015 g CHX and CNC + 0.00015 g CHX compared to the sample without drug (CNC) may be explained by the total dispersion of the dissolved drug in the cellulose solution. 

### 3.9. Drug Release

**Figure 8:**
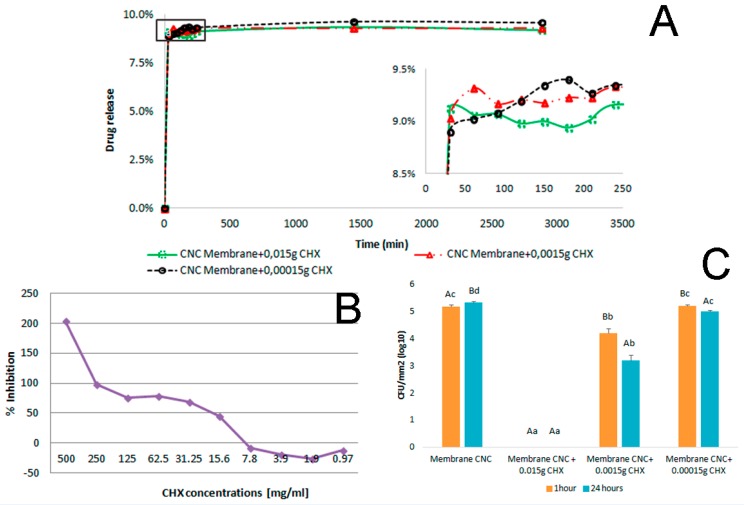
Membrane release profile from 0 to 48 h and highlights for the first 250 min (**A**); in vitro antibacterial activity of chlorhexidine dissolved in DMSO against *S. aureus* (**B**); survival of *S. aureus* ATCC 19095 after modified direct contact test. Different capital letters represent statistically significant differences in the group and different lower case letters represent statistically significant differences between groups (*p* < 0.05) (**C**).

Figure 8A presents the drug release of the membranes as a function of time; results are similar for the three tested concentrations. For the evaluation of drug delivery, the drug was added to CNC solutions and taken to the oven for solidification; this may account for the delivery of CHX around 10%. Literature reports that the mechanisms of adsorption of CHX onto cellulosic fibers appear to be controlled mainly by electrostatic forces between the cationic groups of the CHX and the carboxyl acid groups of cellulose fibers, as well as the hydrogen bonding between the biguanide group and the *p*-chlorophenol of the CHX with hydroxyl groups of cellulose [10]. 

Results demonstrate that the membrane could deliver the antimicrobial drug CHX with a satisfactory performance. The CNC solution proves to be a promising vehicle for drug delivery, considering that the drug is not inserted superficially in the membrane but rather dissolved and inserted during the preparation of the membrane, giving a stronger interaction than membranes which are immersed in the solution containing the drug.

### 3.10. Antibacterial Activity

#### 3.10.1. Minimum Inhibitory Concentration (MIC) and Minimum Bactericidal Concentration (MBC) of Chlorhexidine

Broth microdilution technique was chosen in order to determine the susceptibility of the selected microorganism to the antimicrobial agent studied. The results obtained for the minimum inhibitory concentration and minimum bactericidal concentration are shown in Figure 8B. The results show the MIC and MBC values (31.25 mg·mL^−1^ for *S. aureus*).

The data obtained suggest that the CHX was effective against *S. aureus* at even a 31.25 mg·mL^−1^ concentration. As an expected behavior, the susceptibility of the microorganism varied according to the concentrations of the antimicrobial drugs agent. The inhibition against the microorganism was directly proportional to the concentration of chlorhexidine, constantly increasing as the concentration got higher than 31.25 mg·mL^−1^.

#### 3.10.2. Modified Direct Contact Test (mDCT)

The ability of CNC-CHX membranes to inhibit the growth or even kill *S. aureus* cultivars was evaluated. The CNC membrane without drugs was used as the control, and the inhibition results of *S. aureus* ATCC 19095 are presented in Figure 8C. The CNC membrane + 0.015 g CHX totally inhibited bacterial growth after 1 h and 24 h. There was a proportionally direct decrease in the inhibition of the bacteria growth in accordance with the decrease in drug concentration. In high concentrations, CHX is bactericidal via destruction of the cell membrane. This can be explained because at lower concentrations, CHX has bacteriostatic properties [13].

## 4. Conclusions 

CNCs were successfully obtained by high frequency cavitation induced acid hydrolysis. The analyses were performed to evaluate the characteristics of the particles, and the results showed that all times and temperatures tested are viable to obtain CNC without further treatments. This approach reduces the hydrolysis time by half compared with the conventional hydrolysis (without cavitation) and can be useful when morphology control of the CNC is an important aspect, and thus choice of conditions used in future works depends on the desired morphological features.

In this study, cellulose nanocrystals were applied in solution as excipient obtaining membranes charged with CHX. The methodology for obtaining the membranes proved to be simple and efficient. The results of release experiments showed that the nanocrystal membranes had sustained release properties and the release kinetics show that the quantities delivered are maintained over at least 48 h even with a low concentration of drugs, and this makes CNC membranes a potential candidate for drug delivery applications. The antibacterial activity of the CNC membrane with 0.015 g of CHX was found to be highly effective against *S. aureus*, showing that the CNC-CHX membranes can be used for antimicrobial applications.

In future work, in vitro cytotoxicity assays will be performed to assess the cell viability provided by the CNC-CHX membranes, and the behavior of other drugs also incorporated into CNC membranes will be studied in order to clarify the behavior and compare the membrane with other excipients.

## Figures and Tables

**Table 1:** Chemical composition of the raw material used in this study compared to other sources.

Extractives	Lignin	Hemicelluloses	α-Cellulose
2.58 ± 2.11 ^a^	1.70 ± 0.31 ^a^	15.42 ± 0.53 ^a^	63.91 ± 1 ^a^
1.70 ^c^	24.90 ^b^	13.70 ^b^	73.80 ^b^
-	2.8 ± 0.50 ^c^	8.7 ± 0.20 ^c^	77 ± 0.30 ^c^
-	4.70 ^d^	12.90 ^d^	68.70 ^d^

^a^ (his study); ^b^ [29]; ^c^ [6]; ^d^ [30].

**Table 2:** Crystallinity properties of the different nanocrystals.

Sample/Method	Crystallinity Index (%)		Yield (g/g)	Zeta Potential (mV)	δ_200_ (Å)
	Segal Method	Deconvolution Method			Scherrer Approximated
50-30	86.17	80.19	0.0930	−32.70	54.39
50-45	90.50	85.55	0.1799	−24.30	51.91
50-60	91.63	91.23	0.4032	−24.33	50.68
60-30	90.82	85.88	0.0656	−26.57	53.40
60-45	90.89	87.93	0.1415	−25.40	54.95
60-60	90.90	83.69	0.0808	−31.35	50.01

**Table 3:** Root mean squared roughness (RMS) of the membranes.

SAMPLES	RMS *
CNC + 0.015 g CHX	44.85
CNC + 0.0015 g CHX	28
CNC + 0.0015 g CHX	5.87

* Values were evaluated over an area of 25 nm^2^ at different locations.
